# Romosozumab for managing severe osteoporosis in patients undergoing kidney transplantation: a retrospective case series

**DOI:** 10.1093/jbmrpl/ziaf049

**Published:** 2025-04-14

**Authors:** Ayako Tominaga, Keiji Wada, Yoshiharu Kato, Ken Okazaki

**Affiliations:** Department of Orthopedic Surgery, Tokyo Women’s Medical University, Tokyo, 162-8666, Japan; Department of Orthopedic Surgery, Tokyo Women’s Medical University, Tokyo, 162-8666, Japan; Spine Center, Tomei Atsugi Hospital, Atsugi, Kanagawa, 243-8571, Japan; Kita Shinagawa 3rd Hospital, Tokyo, 140-0001, Japan; Department of Orthopedic Surgery, Tokyo Women’s Medical University, Tokyo, 162-8666, Japan

**Keywords:** chronic kidney disease, BMD, osteoporosis, kidney transplantation, romosozumab

## Abstract

Recipients of kidney grafts often develop severe osteoporosis. However, no consensus has emerged on the most appropriate medications for managing osteoporosis in these recipients. In this study, we investigated the efficacy of romosozumab as an additional treatment option for managing severe osteoporosis in kidney transplant recipients (KTRs). Our retrospective observational study included 12 such recipients who were treated with romosozumab for 12 mo—8 newly initiated on romosozumab and 4 treated with romosozumab after initial treatment with other agents. Endpoints were side effects, new fractures, blood tests, and changes in BMD. Pearson correlation coefficients were used to assess associations of the percent change in bone mineral density after 1 yr of treatment with age, dialysis duration, and time (yr) since transplantation. During treatment with romosozumab, the patients did not develop severe hypocalcemia or experience marked deterioration of kidney function at 1 yr post-treatment. Metabolic markers of bone formation and resorption were similar to those in the general population with osteoporosis. The average changes in BMD at the spine and total hip were 15.18% and 8.83%, respectively, indicating a favorable increase. Further, the change in spine BMD was inversely correlated with age and time since transplantation. Treatment of osteoporosis with romosozumab was observed to be safe for KTRs and had a favorable therapeutic effect on both spine and hip BMD.

## Graphical Abstract

**Graphical Abstract:**
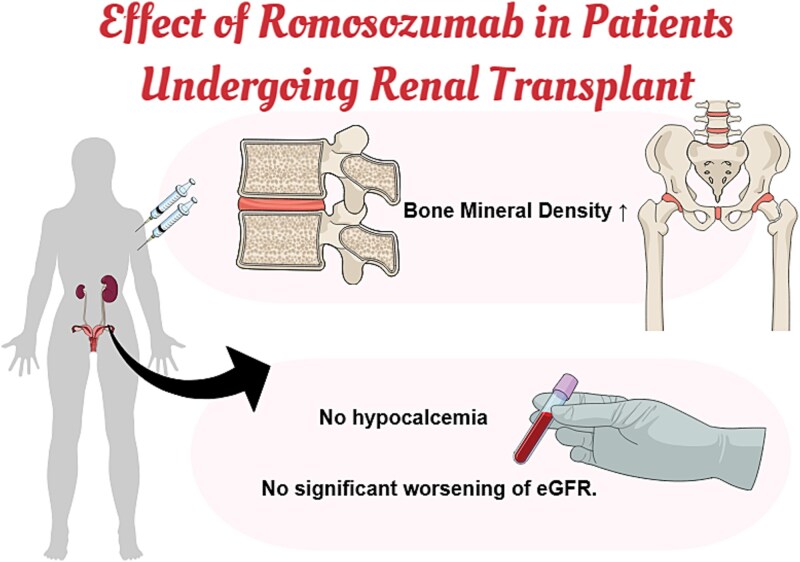

## Introduction

Osteoporosis presents a significant health challenge, and its treatment is difficult.^1^ Similarly, kidney dysfunction is a global health concern, with approximately 10%-13% of various populations affected by CKD.^2^ In the United States, 1 in 7 people are estimated to have CKD, amounting to approximately 37 million patients.^3^ In Asia, overall prevalence rates range from 7% in South Korea to 34.3% in Singapore.^4^ In recent years, patients with chronic kidney failure have been found to have an increased risk of fractures, which increases with worsening kidney function.^5^

As CKD progresses, dialysis or kidney transplantation becomes necessary. Despite the temporal stagnation caused by COVID-19, kidney transplantation in the United States has increased exponentially in recent years, with 26 309 procedures reported in 2022.^6^ Kidney transplant recipients (KTRs) are reported to be at an increased risk of osteoporosis and fractures.^7^ Although affected recipients must receive early and appropriate osteoporosis treatment, no clear consensus has emerged on the appropriate medications for treatment.^8^ Romosozumab, available since 2019, is a potent antiosteoporotic agent that combines bone formation–promoting and bone resorption–inhibiting effects.^9^ Phase III clinical trials have shown positive results in patients with postmenopausal osteoporosis treated with romosozumab for 12 mo, with a 13.3% increase in spine BMD and a 6.8% increase in hip BMD.^9^ Similarly, positive data have been reported in real-world clinical practice.^9^^,^^10^ Romosozumab has been successfully used in patients with chronic kidney failure, those undergoing dialysis, or both; however, no reports have yet been published regarding its use in KTRs.^11^^,^^12^ In this study, we investigated the efficacy of romosozumab as another treatment option for managing severe osteoporosis in KTRs.

## Materials and methods

### Participants

Our retrospective observational study enrolled 14 patients with severe osteoporosis who had undergone kidney transplantation, visited the Department of Orthopedic Surgery at Tokyo Women’s Medical University between 2020 and 2024, and received romosozumab (210 mg per month) for 12 mo. Severe osteoporosis was defined as ≥1 vertebral fracture or fracture of the proximal femur, or a T-score ≤ 2.5 on BMD examination.^13^ Patients with a history of cerebrovascular disease, those who were reintroduced to dialysis before starting treatment, or those who had self-interrupted their treatment were excluded. All patients were treated under the Japanese health insurance system.

### Evaluation of selected clinical and biomedical parameters

The following parameters were evaluated:

▪ Complications, including new fractures during treatment. When patients reported pain during a visit, radiography was used to confirm fractures.
▪ Blood parameters, such as corrected calcium (Ca), phosphorus (P), estimated glomerular filtration rate (eGFR), intact iPTH, and metabolic markers of bone formation (P1NP) and bone resorption (tartrate-resistant acid phosphatase 5b [TRACP-5b]).
  The normal reference ranges for those parameters were as follows:
  Ca: 8.5-10.5 mg/dL^14^;
  P: 2.5-4.5 mg/dL;
  iPTH: 10-65 pg/mL;
  P1NP:
    o Male patients: 18.1-74.1 ng/mL;
    o Female patients (postmenopausal, 45-79 yr): 26.4-98.2 ng/mL.
  TRACP-5b:
    o Male patients: 170-590 mU/dL;
    o Female patients: 120-420 mU/dL.
▪ Percent change in spine and hip BMD.

Blood was collected before romosozumab (month 0), before each subsequent romosozumab injection (months 1, 3, 6, and 9), and at month 13 after all 12 injections were completed. Kidney function pretreatment and post-treatment was evaluated as CKD stages 1-5, based on existing reports^14^ (stage 1, eGFR ≥90 mL/min/1.73 m^2^; stage 2, 60 ≤ eGFR <89 mL/min/1.73 m^2^; stage 3, 30 ≤ eGFR <59 mL/min/1.73 m^2^; stage 4, 15 ≤ eGFR <29 mL/min/1.73 m^2^; and stage 5, eGFR <15 mL/min/1.73 m^2^).

BMD was assessed before and at 6 and 12 mo after treatment using DXA measurements of spinal bone density (the mean of L1-L4), and total hip and femoral neck bone density. The testing was performed using the Lunar iDXA system (GE Healthcare).

In this study, romosozumab was administered without concomitant vitamin D preparations such as eldecalcitol, which could potentially cause kidney damage. All patients enrolled in the study were evaluated for kidney function before and after treatment. As diminished effect of history of other antiosteoporosis treatment before romosozumab treatment has been described, parameters other than kidney function were analyzed only in patients who were newly started on romosozumab treatment (treatment-naïve group: *n* = 8).^10^

All study procedures complied with the 1964 Helsinki Declaration and its subsequent amendments. The study was approved by the Tokyo Women’s Medical University Ethics Committee. Informed consent was obtained from all participants.

### Statistical analysis

Statistical analyses were performed using the Easy R interface (Saitama Medical Center, Jichi Medical University) to the R software application (The R Foundation for Statistical Computing).^15^ The one-sample *t-*test was used to analyze changes in corrected Ca. The associations of the percent change in BMD after 12 mo of treatment with age, dialysis duration, and time since transplantation (yr) were analyzed using the Pearson correlation coefficient. An alpha of less than 0.05 was considered statistically significant.

## Results

Of the 14 patients enrolled, 12 were eligible for analysis (Table 1 and Table S1–3.). Of the 12 patients, 8 were romosozumab-naïve, and 4 had been transitioned to romosozumab from other osteoporosis medications, including 3 from bisphosphonate therapy. The patients who transitioned away from bisphosphonates had all been taking oral alendronate (average eGFR : 26.25 mL/min/1.73 m^2^). Before the initiation of romosozumab treatment, 10 patients were categorized as CKD stage 3, and two as CKD stage 4. Kidney failure was caused by IgA nephropathy in five patients; Table 1 presents the causes of kidney failure in the remaining patients. The mean duration of dialysis before and after transplantation was 6.45 ± 2.64 mo and 13.13 ± 3.63 yr, respectively. To protect the kidney graft, methylprednisolone, tacrolimus hydrate, and mycophenolate mofetil were administered, with doses adjusted based on individual blood draw data. The details of each patient’s prescribed medications are provided in the supplementary data (Table S1–3).

**Table 1:** Baseline clinical and biomedical parameters of the analyzed patients.

**Parameter**	**Value**
**Patients (*n*)**	12
**Osteoporosis treatment history (*n*)**	
** Romosozumab-naïve**	8
** Transitioned from bisphosphonate**	3
** Transitioned from teriparatide**	1
**Mean age^a^ (yr)**	60.33 ± 2.99
**Female sex [*n* (%)]**	10 (83.33)
**Mean BMI^a^ (kg/m^2^)**	20.51 ± 0.72
**Mean eGFR (mL/min/1.73 m^2^)**	38.19 ± 2.80
**CKD stage (*n*)**	
** 3**	10
** 4**	2
**Cause of kidney failure (*n*)**	
** IgA nephropathy**	5
** Type 1 diabetes**	1
** Type 2 diabetes**	1
** Scleroderma**	1
** Alcoholism**	1
** Glomerulonephritis**	1
** Unknown**	2
**Mean 25OHD^a^ (ng/mL)**	16.25 ± 1.41
**Mean spine BMD^a^ (g/cm^2^)**	0.87 ± 0.07
**Mean hip BMD^a^ (g/cm^2^)**	0.62 ± 0.02
**Mean T-score^a^**	
** Spine**	−2.16 ± 0.50
** Hip**	−2.73 ± 0.20
**History of fracture (*n*)**	
** Vertebral**	4
** Proximal femur**	2
** Distal radius**	1

Abbreviation: eGFR: estimated glomerular filtration rate.

a ±SE.

### Complications and fractures

Complications during romosozumab treatment included injection site pain and redness in four patients. One patient sustained a unilateral clavicle fracture and three ipsilateral rib fractures due to high-energy trauma (a fall down stairs during heavy rain). No patient developed a fragility fracture.

### Kidney function

Figure 1-1A presents the eGFR trend during romosozumab treatment. The means ± SE of eGFR at baseline and 12 months of treatment were 38.19 ± 2.80 and 35.93 ± 2.32 mL/min/1.73 m^2^, respectively, indicating maintained eGFR. Two patients experienced a change in CKD stage from baseline to after romosozumab treatment. In one patient, eGFR declined from 34.1 mL/min/1.73 m^2^ at baseline to 27.00 mL/min/1.73 m^2^ at the end of treatment. In another patient, eGFR declined from 30.40 mL/min/1.73 m^2^ at baseline to 28.50 mL/min/1.73 m^2^ at the end of treatment.

**Figure 1-1:**
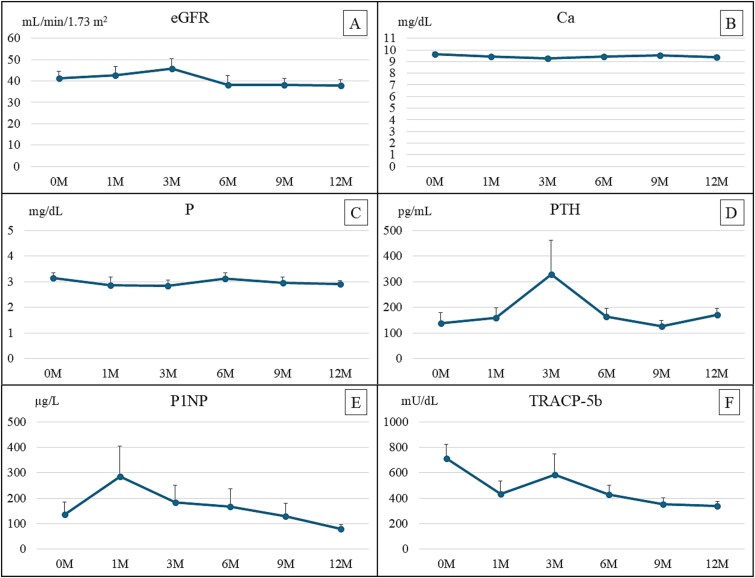
(A) Trend of the mean (±SE) estimated glomerular filtration rate (eGFR) during romosozumab treatment. The value at treatment initiation was 38.19 ± 2.80 mL/min/1.73 m^2^, and after 12 mo of treatment, it was 35.93 ± 2.32 mL/min/1.73 m^2^, indicating eGFR maintenance. (B) Corrected calcium (Ca) during romosozumab treatment. Ca reached its lowest value at the third month of treatment. (C) Mean (±SE) phosphorus (P) during romosozumab treatment. P remained constant throughout the 12 mo of treatment. (D) Mean (±SE) intact PTH during romosozumab treatment. Intact PTH reached its highest value in the third month of treatment, parallelling Ca. (E) Mean (±SE) total P1NP during romosozumab treatment. This bone formation marker increased rapidly in the first month after treatment initiation and then gradually declined. (F) Mean (±SE) tartrate-resistant acid phosphatase 5b (TRACP-5b) during romosozumab treatment. This bone resorption marker transiently increased in the third month after treatment initiation, but declined overall during treatment, remaining below baseline throughout 12 mo of treatment.

### Corrected Ca, P, and iPTH

Figure 1-1B-D presents changes in Ca, P, and iPTH during romosozumab treatment. Ca levels changed significantly but remained within the reference range, and no major deviations observed in any patient. The lowest Ca levels, coupled with the highest iPTH levels, were observed at 3 mo of romosozumab treatment, while P remained constant throughout. No patient experienced severe hypocalcemia, and blood Ca was managed with observation without active correction.

### Metabolic markers of bone formation and resorption

Figure 1-1E and F presents the time course of P1NP and TRACP-5b during romosozumab treatment. P1NP increased rapidly during the first month after treatment initiation and gradually declined thereafter. TRACP-5b increased transiently at 3 mo after treatment initiation, then declined throughout the course of treatment, remaining below baseline at every time point thereafter.

### Percent change in BMD

Figure 1-2A-C presents the percent change in spine, total hip, and femoral neck BMD during romosozumab treatment. Spine BMD increased from 10.11% ± 2.57% at 6 mo to 15.18% ± 4.09% at 12 mo. Similarly, total hip BMD increased from 3.79% ± 1.22% at 6 mo to 8.83% ± 2.36% at 12 mo, and femoral neck BMD increased from 3.27% ± 2.14% at 6 months to 6.34% ± 3.77% at 12 mo.

**Figure 1-2:**
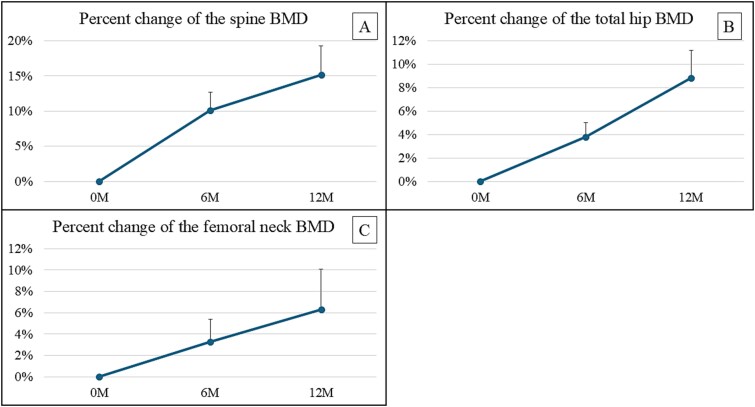
(A) Percent change in spine BMD during romosozumab treatment. Spine BMD rose to 15.18% ± 4.09%. (B) Percent change in total hip BMD during romosozumab treatment. Total hip BMD rose to 8.83% ± 2.36%. (C) Percent change of femoral neck BMD during romosozumab treatment. Femoral neck BMD rose to 6.34% ± 3.77%. All these BMD changes were favorable for the patients.

**Figure 2:**
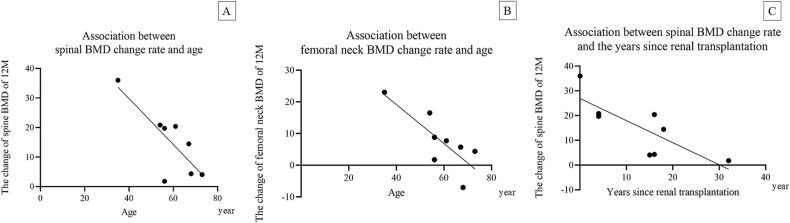
(A) The percent change in spine BMD was significantly inversely correlated with age (correlation coefficient: *r* = −0.7788 [equation: Y = −07788 ^*^ X + 60.94]; 95% confidence interval [CL]: −1.381 to −0.1763; *p* = .02). (B) The percent change in femoral neck BMD was significantly inversely correlated with age (correlation coefficient: *r* = −0.6153 [equation: Y = −0.6153 ^*^ X + 43.77]; 95% CI: −1.089 to −0.1421; *p* = .019). (C) The percent change in spine BMD was inversely correlated with years since kidney transplantation (correlation coefficient: *r* = −0.8927 [equation: Y = −0.8927 ^*^ X + 26.90]; 95% CI: −1.576 to −0.2091; *p* = .019).

The percent change in spine and femoral neck BMD were significantly inversely correlated with age (spine: *r* = −0.7788 [equation: Y = −07788 ^*^ X + 60.94], 95% confidence interval [CI]: −1.381 to −0.1763, *p* = .02; femoral neck: *r* = −0.6153 [equation: Y = −0.6153 ^*^ X + 43.77], 95% CI: −1.089 to −0.1421, *p* = .019; Figure 2A and B). Additionally, the percentage in spine BMD was significantly inversely correlated with the number of years since kidney transplantation (*r* = −0.8927 [equation: Y = −0.8927 ^*^ X + 26.90], 95% CI: −1.576 to −0.2091, *p* = .019; Figure 2C). No clear correlation between dialysis duration and the rate of change in BMD was observed.

## Discussion

In KTRs, romosozumab treatment was associated with significant increases in both spine and hip BMD, without significantly affecting kidney function or causing notable complications. Recent studies have shown that patients with chronic kidney failure have an increased risk of bone fractures, which worsens as kidney function declines.^5^ Abnormalities in bone metabolism in patients with chronic kidney failure—called CKD mineral and bone disorder—are caused by an imbalance between bone formation and resorption as kidney function deteriorates.^5^^,^^16^ The use of corticosteroids, immunosuppressive drugs, a history of dialysis, and diabetes mellitus further increases the risk of osteoporosis and fractures in KTRs.^7^ In particular, KTRs have a 5-fold increased risk of proximal femur fractures compared with the general population.^7^ The most appropriate medication for treating osteoporosis in KTRs is yet to be determined.^8^ In the present study, we aimed to expand the range of medications for KTRs with severe osteoporosis.

Bisphosphonates and denosumab are commonly used for treating osteoporosis in KTRs. Bisphosphonates have been observed to increase vertebral BMD in treated KTRs compared with untreated patients,^17^ while denosumab has been reported to significantly increase vertebral and hip bone density in KTRs compared with control groups.^18^ However, the percent change in BMD has been reported to range between 0% and 4%, a relatively weak effect.^17^^,^^18^

Previous reports on romosozumab treatment in patients with kidney failure have observed favorable increases in BMD and decreases in fracture risk, even in patients with chronic kidney failure, with no apparent nephrotoxicity.^19^^,^^20^ Furthermore, recent reports have shown that romosozumab can be safely and effectively used in hemodialysis patients.^12^^,^^21^ Being a monoclonal antibody that is primarily and proteolytically degraded in the liver and intraretinal system, romosozumab is not expected to directly affect kidney function.^19^^,^^21^ Previous studies have recommended maintaining the dose of romosozumab even in cases of impaired kidney function.^21^ Although patients with moderate chronic kidney failure rarely develop severe hypocalcemia, some studies have observed markedly low blood levels of Ca in patients receiving romosozumab and undergoing dialysis, suggesting a need for caution.^12^^,^^19^

In this study, no marked decrease in Ca levels or severe hypocalcemia was observed. In a general population receiving romosozumab, Ca levels were observed to be lowest at 3 mo after treatment initiation, which accords with the Ca dynamics we observed in KTRs.^10^ Compared with patients undergoing dialysis, KTRs might have a lower risk of hypocalcemia. Testing Ca levels before and at 3 mo after treatment initiation might be advisable.

With respect to markers of bone metabolism, a characteristic of romosozumab kinetics is a transient elevation in bone formation markers immediately after treatment initiation.^9^^,^^10^ This characteristic was also observed in the KTRs in our study, demonstrating that the kinetics did not significantly differ from those in the general population.^9^^,^^10^ Similarly, in the KTRs in our study, bone resorption markers declined in the first month after treatment initiation and slightly increased in the third month before decreasing again, which is not significantly different from the kinetics in the general population.^9^^,^^10^

A previous report also indicated a slight increase in TRACP-5b at 3 mo but made no detailed assessment.^22^ Furthermore, no other studies have discussed that marker. We hypothesize that the serum Ca nadir at 3 mo, which triggers a simultaneous increase in iPTH, likely induces a subsequent increase in TRACP-5b.

With regard to BMD, a phase III clinical trial of romosozumab reported favorable results in postmenopausal women with osteoporosis, with a 13.3% increase in spinal BMD and a 6.8% increase in hip BMD.^9^ We observed similarly favorable results in KTRs, who experienced increased spine and hip BMD. However, unlike the postmenopausal women, KTRs have bone fragility because of abnormal bone quality; thus, the extent to which increased BMD will reduce their fracture risk remains unclear. Nevertheless, considering that BMD remains the mainstay measure of osteoporosis for evaluating drugs to improve bone quality, we consider that a favorable increase in BMD might contribute to a decrease in fracture risk.^5^^,^^7^^,^^16^

In our study, the percent change of BMD in KTRs was observed to correlate with age and time since transplantation. Further research is needed to determine the factors that most contribute to BMD, particularly spinal BMD. Further studies with more patients are needed for multiple regression analyses. Patients who have undergone kidney transplantation should be evaluated for osteoporosis as early as possible. In KTRs who develop osteoporosis, drug efficacy will increase with early therapeutic intervention.

Steroid therapy in combination with other medications is common after kidney transplantation. In long-term steroid therapy, apoptosis of osteoblasts and osteocytes, leading to decreased bone formation, has been reported (Chotiyarnwong. P., et al, 2020, Nat Rev Endocrinol.). Steroids also prolong the lifespan of osteoclasts, resulting in increased bone resorption.^23^ Thus, steroids are known to suppress bone formation (Chotiyarnwong.P., et al, 2020, Nat Rev Endocrinol.). Previous studies have reported that romosozumab significantly improved BMD even in patients with rheumatoid arthritis receiving steroid therapy.^23^ That finding accords with the results of our study.

### Limitations

This retrospective observational study with a small sample size used data obtained from electronic health records for a single ethnic group, thus presenting inherent limitations. Age and sex biases were also present in this study. Several studies, including ours, have reported the effects of romosozumab in patients with kidney impairment, but the small sample size limits the generalizability of the findings. Moreover, the absence of a control group makes it difficult to attribute improvements in BMD solely to romosozumab. Previous studies have reported limited improvements in Ca and P metabolism and in serum fibroblast growth factor 23 after kidney transplantation, but no studies have documented recovery from osteoporosis or improvements in BMD.^24^ The significant increase in BMD within 12 mo, as observed in this study, could therefore likely be primarily attributed to the effects of romosozumab. In our patients, serum 25OHD was below the reference range. However, the transplant surgeons indicated that the number of vitamin D–related medications should be minimized to protect the kidney graft. In addition, eldecalcitol has been reported to be a potential cause of hypercalcemia, with an incidence rate varying widely from 0.88% to 21%, depending on the report. Patients with impaired kidney function seem to be particularly prone to this condition. Cases of eldecalcitol-induced hypercalcemia leading to acute kidney injury have also been reported, with a frequency of 46.4%. Active vitamin D preparations were therefore avoided in this study cohort. Given that other antiosteoporosis drugs affect kidney function, future research on transition therapy beyond romosozumab is needed.

## Conclusions

Romosozumab is associated with significant increases in BMD, even in patients with secondary osteoporosis, such as those who have undergone kidney transplantation. In this study, the use of romosozumab in KTRs resulted in mild hypocalcemia in just one patient during the 12-mo follow-up. The mean eGFR in the study cohort was 38.19 ± 2.80 mL/min/1.73 m^2^ before treatment and an almost stable 35.93 ± 2.32 mL/min/1.73 m^2^ after treatment. These study results expand the range of available medications for treating osteoporosis in KTRs at high risk of bone fractures.

## Supplementary Material

Table_AYATTJ-19_MI-5_Supp_ziaf049

## Data Availability

The data underlying this article will be shared upon reasonable request to the corresponding author. Patient consent statement This is a case report, and written informed consent was obtained from the patient for the publication of this report.
